# Biomechanical assessment of vulnerable plaque: from histological evidence to ultrasound elastography and image-based computational patient-specific modelling

**DOI:** 10.3389/fbioe.2025.1478408

**Published:** 2025-03-07

**Authors:** Nicoletta Curcio, Michele Conti, Rosanna Cardani, Laura Valentina Renna, Giacomo Dell’Antonio, Vlasta Bari, Giovanni Nano, Giulia Matrone, Daniela Mazzaccaro

**Affiliations:** ^1^ 3D and Computer Simulation Laboratory, IRCCS Policlinico San Donato, San Donato Milanese, Italy; ^2^ Department of Civil Engineering and Architecture, University of Pavia, Pavia, Italy; ^3^ Biobank BioCor, IRCCS Policlinico San Donato, San Donato Milanese, Italy; ^4^ Unit of Pathology, Cerba Healthcare Laboratories, Limena, Italy; ^5^ Department of Cardiothoracic, Vascular Anesthesia and Intensive Care, IRCCS Policlinico San Donato, San Donato Milanese, Milan, Italy; ^6^ Department of Biomedical Sciences for Health, University of Milan, Milan, Italy; ^7^ Operative Unit of Vascular Surgery, IRCCS Policlinico San Donato, San Donato Milanese, Italy; ^8^ Department of Electrical, Computer and Biomedical Engineering, University of Pavia, Pavia, Italy

**Keywords:** carotid plaque vulnerability, biomechanical assessment, non-invasive imaging, elastography, patient-specific plaque modelling, finite element analysis

## Abstract

The assessment of carotid plaque vulnerability is a relevant clinical information that can help prevent adverse cerebrovascular events. To this aim, in this work we study the ability of different non-invasive methods for assessing plaque vulnerability in patients undergoing carotid endarterectomy (CEA). Histological examinations of patients’ plaque samples were conducted after CEA while ultrasound (US) and computed tomography angiography (CTA) acquisitions were performed preoperatively. US acquisition included point shear wave elastography (p-SWE) and a radio frequency echo-based wall tracking mode for the evaluation of arterial wall stiffness. CTA images were segmented, and the results were used for an *ad hoc* procedure that semi-automatically reconstructed the atherosclerotic wall providing a 3D model of the different plaque components to perform patient-specific finite element analysis (FEA) of stress distributions. One hundred patients were involved in the study and a macroscopic assessment of the surgeon was used to classify carotid atherosclerotic plaques as vulnerable or stable. The data derived from histological analysis, US acquisitions and FEA were correlated with the outcome of the classification. Indeed, histological features differentiated between vulnerable and stable plaques, confirming the surgeon’s classification. From p-SWE, the measurement of Young’s Modulus (YM) in stable plaques was significantly higher than in vulnerable plaques. Also stress indexes related to the Von Mises and Max Principal stresses from FEAs showed statistically significant differences between plaque groups. These results demonstrate that both stiffness-related US measurements and stress parameters derived preoperatively from computational analyses were able to differentiate patients with vulnerable plaques from ones with stable plaques. Thus, the development and application of new methods for a non-invasive biomechanical assessment of atherosclerotic artery walls could give valuable information for plaque vulnerability evaluation.

## 1 Introduction

Stroke is the main cause of long-term disability and the third leading cause of death (Kolos et al., 2015), and carotid artery (CA) stenosis due to atherosclerosis accounts for a major cause of all ischemic strokes (Banerjee and Chimowitz, 2017). Atherosclerosis is a pathological condition characterized by the accumulation of inflammatory cells, lipids, extracellular matrix, and other materials within the inner layers of artery walls, which leads to the development of an atherosclerotic plaque and the consequent narrowing of the arterial lumen. Plaques that cause a severe narrowing of the lumen increase the risk of having cerebrovascular events. In such cases, current guidelines recommend surgical intervention to prevent stroke (Spence, 2016). Carotid endarterectomy (CEA) has been reported as a safe and effective procedure to reduce stroke risk by removing the atherosclerotic plaque from the CA, especially for symptomatic patients.

Nevertheless, some studies highlighted that plaques can cause severe cardiovascular events regardless of the degree of stenosis (Kashiwazaki et al., 2019; Truijman et al., 2014). Clinical practice, in fact, suggests that the type of plaque is a crucial determinant of stroke risk for the same degree of stenosis, as certain plaques are more susceptible to inflammation and rupture and, therefore, more likely to cause cerebrovascular events (Mughal et al., 2011). Such plaques are defined “vulnerable.” From the histopathological point of view, they are characterized by active inflammation, a large lipid core, thin fibrous cap and may have features of intraplaque haemorrhage, neovascularization, necrosis, small calcification and surface ulceration. Currently, the identification of plaque vulnerability features can only be performed through histological examination after the plaque has been surgically removed (Gerosa et al., 2023).

Since modern literature questions the benefit of CEA in asymptomatic patients with optimal medical treatment (Spence, 2016), it appears crucial to identify a method suitable for the preoperative phase to offer plaque information as accurately as the histological analysis performed after CEA. Indeed, preoperative identification of vulnerable plaques would enable the identification of a high-risk subgroup of patients who could mostly benefit from a surgical procedure, thereby allowing for *in vivo* risk stratification.

The underlying mechanism for plaque rupture has not been fully understood yet. Biomechanical factors, such as structural and hemodynamic stresses on the atherosclerotic vessel wall, are among risk factors that promote the progression and compositional changes of atherosclerotic plaques (Brown et al., 2016). Furthermore, it has been hypothesized that sudden rupture is triggered by local stress concentrations, when structural stress exceeds the plaque strength (Teng et al., 2015). Consequently, biomechanical features can be considered crucial in plaque vulnerability assessment.

Several different medical imaging modalities, such as computed tomography angiography (CTA), magnetic resonance angiography (MRA), ultrasonography and positron emission tomography (PET), were employed to assess morphological characteristics of CA wall and plaque in a three-dimensional (3D) way. However, these methods neither detect and distinguish all the different plaque components nor provide information related to biomechanical assessment of tissues.

Recently, ultrasound (US) elastography has been increasingly used as a non-invasive imaging technique, that could help to obtain additional information on carotid artery stiffness and thus on its composition, possibly improving the assessment of plaque rupture (Bulum et al., 2022). Different US elastography techniques exist, based on the type of physical quantity measured or on the type of force applied to deform the underlying tissues. Quasi-static methods (also called strain elastography) use a manual compression on the skin and quantify the percentage of tissue strain, which produces a deformation map. Dynamic methods instead use an acoustic radiation force impulse (ARFI) or a mechanical vibrator to generate shear waves which propagate through the tissues; thus, they are also known as shear wave elastography (SWE). Since the velocity of the shear waves is measurable and proportional to tissue elasticity, quantitative values of tissue stiffness can be determined in terms of Young’s modulus (YM). A specific modality of SWE is the point-SWE, which provides an averaged YM measure within a predefined region of interest (ROI). Since the stiffer the tissue, the higher the YM, vulnerable plaques exhibit lower YM, reflecting changes in deformation and stress/strain characteristics.

Another promising method for noninvasively studying plaque biomechanical characteristics is the use of computational simulation models and finite element analysis (FEA). Computational simulations allow for the computation of 3D stress/strain distributions in patient-specific diseased arteries of various types and morphologies, which could be used to estimate the risk of plaque rupture.

The assessment of plaque vulnerability has been considered in several previous works that studied different vessel types, stress values, and material models by performing computational analyses integrating different imaging techniques (Holzapfel, 2014). The principal findings from FEA studies regard: (i) the association between stress and plaque rupture (Leach et al., 2010; Teng et al., 2015; Costopoulos et al., 2017; Warren et al., 2022) or cerebrovascular events (Sadat et al., 2010); (ii) the factors that impact on the stress distribution (Gao and Long, 2008; Gao et al., 2009; Akyildiz et al., 2011); (iii) the influence of mechanical forces on the progression of the lesions (Tang et al., 2013; Timmins et al., 2016; Kang et al., 2016; Liu et al., 2017).

In this paper, we aimed to evaluate the role of ultrasound point SWE (pSWE) and of computational FEA in assessing the biomechanical features of vulnerable and stable plaques in a group of asymptomatic patients submitted to CEA for a significant carotid stenosis, correlating the results obtained from these methods to those of the macroscopic and microscopic analysis of the excised plaques.

The paper is organized as follows: in Section 2 we illustrate the procedures for clinical data acquisitions, histological analyses and 3D FEAs of patient-specific models derived from CTA images; in Section 3 we report the outcomes of analyses on the collected data; in Section 4 the results are discussed and compared to previous works, the potentialities and limitations of our study are illustrated, as well as possible future developments; Section 5 finally provides the conclusions.

## 2 Materials and methods

### 2.1 Patients’ recruitment and data acquisition

This prospective monocentric study was approved by the Ethics Committee of San Raffaele Hospital on 20 June 2019 (110/int/2019) and registered on ClinicalTrial.gov (ClinicalTrials.gov Identifier: NCT05566080). All patients enrolled in the study at IRCCS Policlinico San Donato gave written informed consent. The study involved the enrolment of 100 patients who underwent CEA at the Vascular Surgery Unit of IRCCS Policlinico San Donato due to the presence of atherosclerotic plaques, causing asymptomatic critical stenosis, according to the current guidelines. For each enrolled patient, demographic data such as sex, age, body max index (BMI), and cardiovascular risk factors including the presence of dyslipidaemia coronary artery disease (CAD), chronic obstructive pulmonary disease (COPD) history, diabetes and history of previous cerebrovascular events were collected.

All patients underwent a preoperative evaluation of carotid stenosis using US imaging and CTA.

The vascular surgeon (DM) also provided a visual macroscopic assessment of the removed plaques of the enrolled patients, in order to classify them as vulnerable or stable plaques. Plaques with prevalence of calcifications were classified as stable plaques; meanwhile, plaques with soft components were classified as vulnerable.

### 2.2 Histology analysis

All carotid plaques were removed in one piece. Immediately after removal, collected plaques were transferred to the Biobank BioCor where a tissue sample approximately 5 mm thick, obtained by a cross-sectioning of a representative part of the plaque was fixed with 10% buffered formaldehyde. Samples were then processed using a routine paraffin technique, some of them were decalcified by a hydrochloric acid solution. Five-micron-thick parallel sections were cut, deparaffinised in xylene and hydrated in graded alcohol. Sections were stained with haematoxylin and eosin or by an indirect immunohistologic method to detect macrophages (CONFIRM anti-CD68 (KP-1) mouse primary antibody and CD163 (MRQ-26) mouse monoclonal primary antibody; Roche Diagnostic).

An experienced pathologist (GDA) evaluated the histological slides using a bright-field optical microscope Olympus BX43.

Since atherosclerosis was defined as the presence of intimal plaques composed by deposits of lipids and proliferating spindle cells, fragmentation of elastic lamina, degeneration of smooth muscle, medial calcification, adventitial fibrosis, and chronic inflammatory infiltration, these different features were considered and classified by the pathologist using a semi-quantitative grade scale, as recommended by (Lovett et al., 2004). More specifically, in our study the following features were considered: the presence of a necrotic/haemorrhagic core, the fibrous cap thickness, the presence of inflammatory infiltrate, the composition of the atheroma and positivity for CD68 and CD163. The corresponding scores were calculated by the pathologist as follows:

o presence of atheroma/necrosis: score = 0 if less than 10%, score = 1 if between 10% and 50%, score = 2 if greater than 50%;
o fibrous cap thickness: score = 3 if < 200 μm, score = 2 if in the range 200–400 μm, score = 1 if in the range 400–800 μm, score = 0 if > 800 µm;
o presence or absence of inflammation: score = 1 for inflamed plaque, score = 0 for non-inflamed plaque;
o presence (score = 1) or absence (score = 0) of cholesterol;
o immunohistochemical response to CD163 and CD68: score = 0 if there is no response, score = 1 if the positive response was up to 5% of the plaque area, score = 2 if between 5% and 10%, and score = 3 if greater than 10%.

In all cases, a lower score indicated a more stable plaque, whereas a higher score indicated a more vulnerable plaque. About 10% of cases were blind revised or underwent further sections.

### 2.3 Ultrasound image acquisition

The patients were scanned before undergoing CEA for asymptomatic stenosis. In particular, the US acquisitions were conducted by experienced vascular surgeons using a MyLab Eight scanner and a 7.5 MHz linear probe, model L4-15 (both from Esaote S.p.A., Genova, Italy). The acquisitions were performed with the patients in a supine position and with a slight neck extension, on the side of the carotid stenosis. The device was equipped with two different software packages, i.e., Quality Arterial Stiffness (QAS) and q-Elaxto modalities.

**FIGURE 1:**
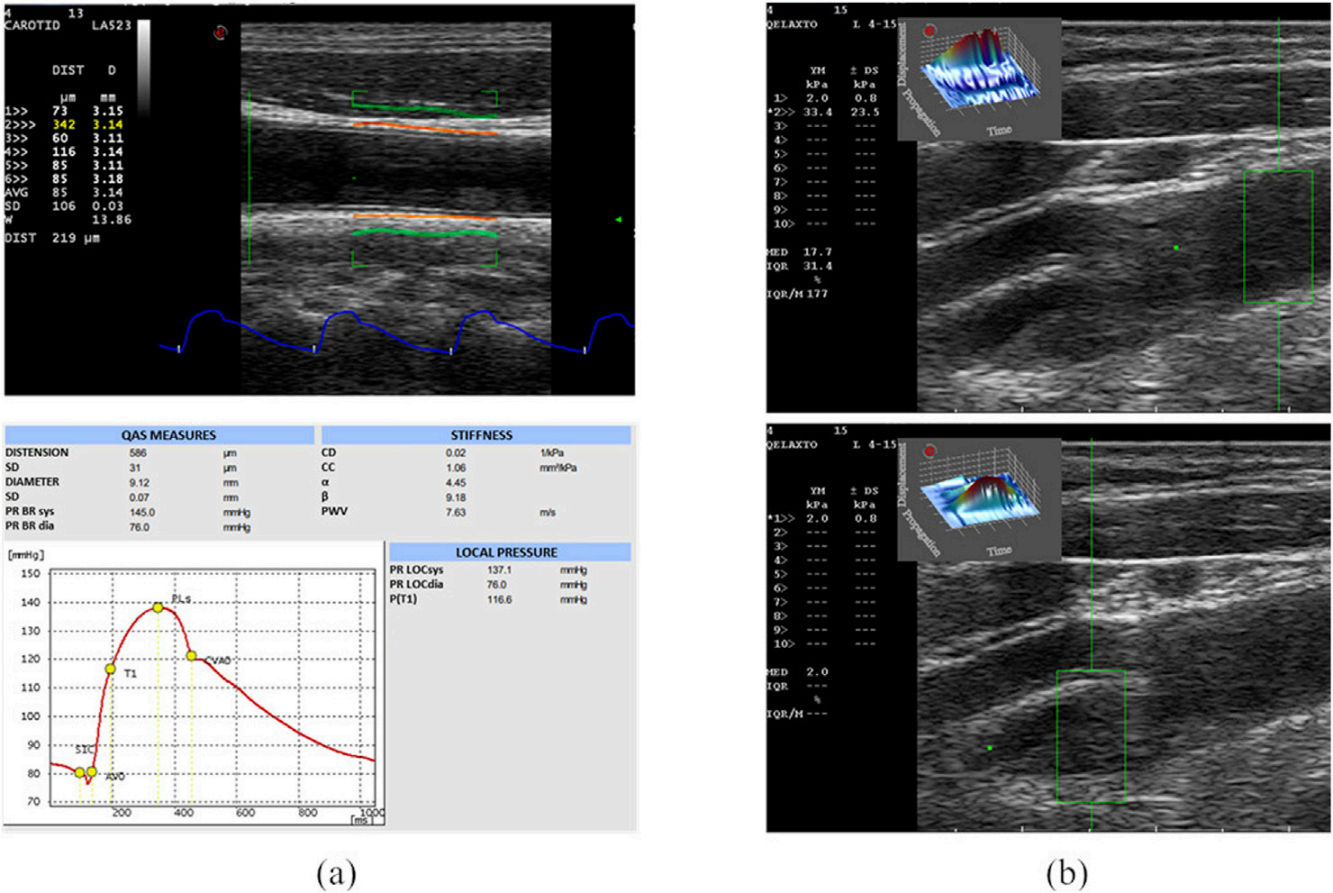
US acquisitions. On the left **(A)**, the QAS modality acquisition and the obtained report are represented. On the top, the B-mode image of the CCA, on which the vessel distension waveform is extracted (in blue) using the QAS modality (Esaote), is shown. The QAS mode automatically detects the vessel wall average diameter (in orange) and its consequent amplified movement (in green). Each acquisition provides a report (on bottom) including the computed parameters and the plot of the local pressure waveform. From the report, we analyzed the following computed parameters [expressed as means and standard deviation (SD)]: Distension, Diameter, Distensibility coefficient (DC), Compliance coefficient (CC), alpha stiffness (α), beta stiffness (β), Pulse Wave Velocity (PWV). On the right **(B)**, the B-mode images of the diseased CA, on which the clinician sets the ROI (in green) for pSWE, are shown. The top image shows the YM measure in a region without plaque, while the bottom image shows the YM measure in the plaque.

QAS derives carotid pressure waveforms by implementing a radiofrequency (RF) echo-based tracking of arterial wall distension (Meinders and Hoeks, 2004; Vermeersch et al., 2008; Esaote, 2018). In this case, the linear probe was placed along a longitudinal axis at 1 cm down from the common carotid artery (CCA) bifurcation, strictly perpendicular to the ultrasound beam, with both walls clearly visualized. An automatic real-time measurement of the change in diameter of the vessel walls between the systolic and diastolic phases was performed, plotting the instantaneous distension waveform (see Figure 1A). The local CA pressure waveform was calculated from the patient’s diastolic and systolic brachial pressure values and arterial cross-section values derived from the distension waveform. Therefore, the software automatically calculated the following measurements, starting from distension and pressure waveforms, which were then provided in a report (Figure 1A):

- Distension (the average of six successive measurements);
- Diameter (the average of six successive measurements);
- Distensibility coefficient (DC), i.e., the absolute change in vessel diameter during systole for a given pressure change;
- Compliance coefficient (CC), i.e., the relative change in vessel diameter during systole for a given pressure change;
- Alpha stiffness (α), which is the elastic coefficient of the vessel;
- Beta stiffness (β), which is the elastic coefficient normalized on the diameter;
- Pulse-wave Velocity (PWV).

The same probe was also used in the q-Elaxto modality, which performs a pSWE acquisition, creating a localized perturbation in the ROI around the focused US beam. This “pushing” phase is followed by a tracking phase, which provides a quantitative estimate of stiffness inside the ROI, i.e., the tissue’s average YM, in kPa, in the selected region. The linear probe was placed longitudinally on the side of the carotid and the acquisitions were performed on the distal and proximal part of the plaque region, obtaining a measure of the YM of the plaque and two measure of the YM of the CCA and internal carotid artery (ICA) walls, as shown in Figure 1B.

### 2.4 Computational analysis

The following sections explain the workflow used to quantify the stress distribution of an atherosclerotic CA by means of 3D patient-specific vessel modelling and structural simulations derived from CTA scans. The developed 3D geometric modelling methodology and the simulation settings are described in detail in Curcio et al. (2023).

#### 2.4.1 3D patient-specific modelling of the vessel and plaque

The segmentation of the DICOM sequences of CTA scans was performed by using a semi-automatic method implemented in ITK-Snap (www.itksnap.org), or by manual reconstruction when plaque components were not easily detectable in the images.

**FIGURE 2:**
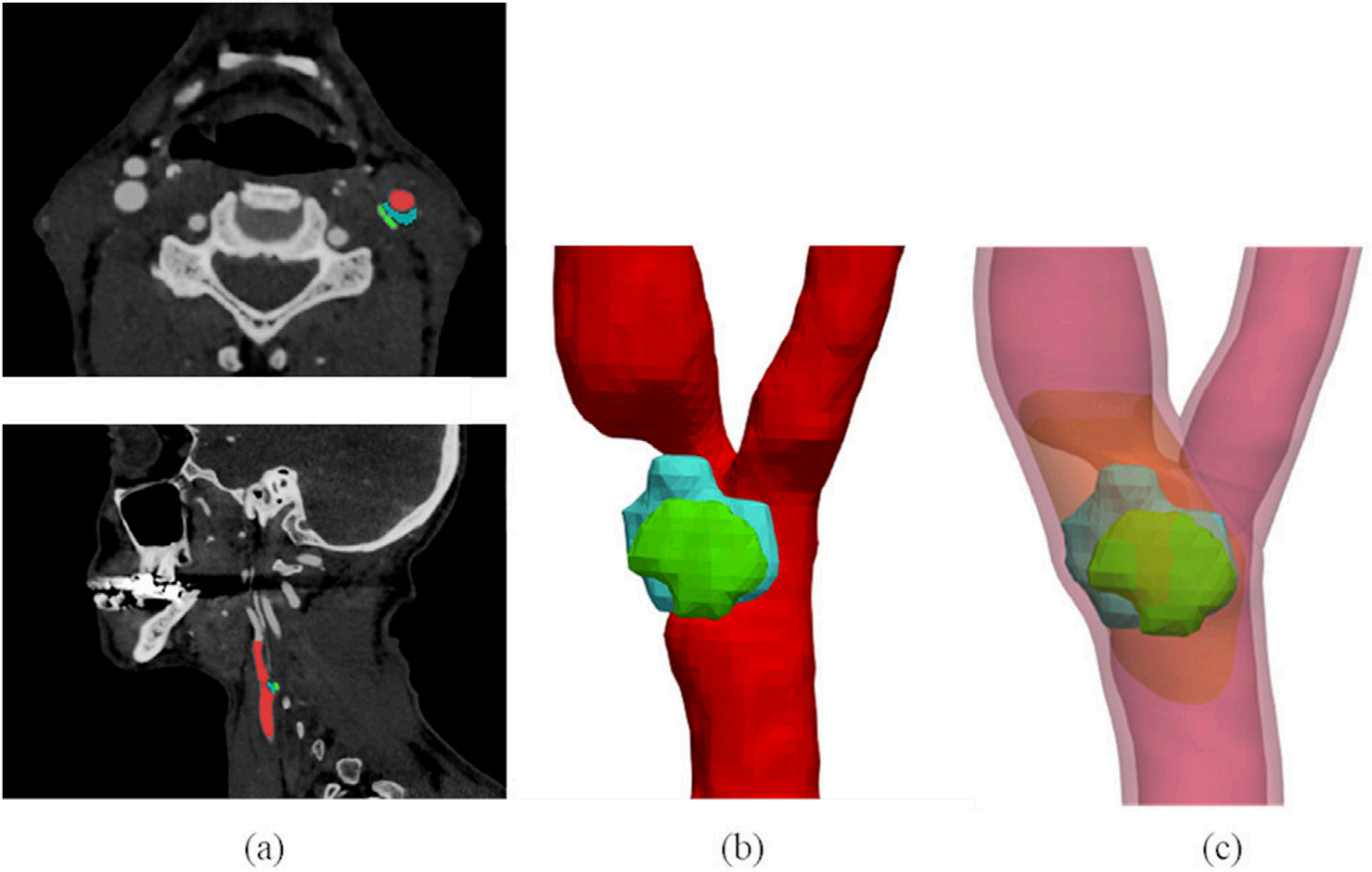
3D plaque model reconstruction from CTA images. CTA axial and sagittal views **(A)**, and corresponding segmentation results **(B)**: the lumen is labelled in red, the lipid content of the plaque is represented in blue and the calcific content in green. Final atherosclerotic wall geometry **(C)** obtained from the corresponding segmentation, by enlarging the lumen sections to include calcific and lipid components of the plaque in the CA walls, and by filling the stenotic region (where other components of plaque are not present) with the fibrous content. The healthy wall is labelled in light red, the fibrous content of the plaque is represented in orange. The figure is adapted from (Curcio et al. 2023).

CTA segmentation allows to obtain the CA lumen and calcific or lipidic plaque component geometries. The CA wall and the plaque fibrous component, instead, are usually not clearly distinguishable in CTA images, and, for this reason, they were automatically reconstructed after image segmentation. The detailed description of the developed reconstruction procedure can be found in our previous study (Curcio et al., 2023) and is illustrated in the Supplementary Material too. This method allows reconstructing the patients’ atherosclerotic carotid walls, starting from lumen and plaque segmentations, with an *ad hoc* developed semi-automatic procedure, while it automatically generates the model of the plaque fibrous component. In this way, it is possible to model more accurately an atherosclerotic vessel by considering different plaque compositions, as illustrated in Figure 2.

Thanks to 3D modelling of the plaque different contents, the volume of each component was computed to perform the morphological analysis. Both absolute and relative volumes were evaluated.

#### 2.4.2 Simulation settings

Finite element simulations based on models generated from CTA images and geometrical reconstructions were performed using Abaqus CAE (Simulia, Providence, RI, United States). Models were simulated as static analyses. The optimum mesh density was obtained by means of a previous convergence analysis. The plaque and arterial wall were assumed to be nearly incompressible, isotropic, linear elastic, with a 0.49 Poisson’s ratio; instead, a different Young’s Modulus was considered for each of them. The used material properties are reported in Table 1 and were obtained as an average of values available in the literature (Lee et al., 1991; Holzapfel et al., 2004; Barrett et al., 2009; Gao et al., 2009; Leach et al., 2010; Mahmoud, Hassan, and Mahmoud, 2019; Djukic et al., 2020; Benitez et al., 2021; Bennati et al., 2021).

**TABLE 1:** Material properties set in FEAs.

Material	Young’s modulus (kPa)
Plaque calcific component	20,000
Plaque lipid component	4
Plaque fibrous component	400
CA healthy wall	550

Blood pressure was considered as uniformly applied to the lumen surface, setting the patient-specific differential carotid pressure obtained from QAS US modality as the blood vessel load. Boundary conditions were applied to fully constrain the distal ends of the bifurcation and the proximal end of the CCA.

#### 2.4.3 Post-processing of simulation results

For each analysed patient, different values of simulated stresses were calculated from the structural simulations of the vessel. Specifically, von Mises stress (VM) and Max Principal stress (MPS) were evaluated, as done in similar computational studies (Gao et al., 2009; Leach et al., 2010; Mahmoud, Hassan, and Mahmoud, 2019; Djukic et al., 2020; Benitez et al., 2021; Bennati et al., 2021), in each component of the plaque. In both cases, the stress value corresponding to 99% of the cumulative volume of the respective plaque component (denoted as VM_99_ and MPS_99_, respectively) and the average stress value within each component (denoted as VM_mean_ and MPS_mean_, respectively) were calculated.

Additionally, these stress values associated with each single component were used to calculate an overall plaque stress index. This index was obtained by linearly combining the corresponding stress values of the three different components, considering the relative volume of each component as a weight. For example, the VM_99_ index is the weighted average of the VM_99_ values in each plaque component, based on their relative volumes.

### 2.5 Statical analysis

Data were analysed using the statistical software SPSS (IBM Corp., Armonk, NY, United States). The Shapiro-Wilk test tested the normality of the distribution of values, and the variables sample was defined as non-normally distributed. The Mann-Whitney U test was used to evaluate the differences in results between the group of patients with a vulnerable plaque and the ones with a stable plaque, as defined by the surgeon. P-values < 0.05 were considered statistically significant.

## 3 Results

In this section, results from statistical analyses on the previously presented parameters are presented. In all tables presented in this section, continuous data values are properly summarized as median with interquartile range (IQR); categorical data are instead presented as numbers with the corresponding percentages.

Demographic data were collected from all 100 patients enrolled in the clinical study, as well as US imaging data. On the other hand, 11 patients had to be excluded from histological analysis because of not complete lumen or wall presence or cutting problems due mainly to artifacts or calcifications. Besides, the CTA segmentation of lumen and plaque components was not performed on four patients’ acquisitions. This was due to the exclusion of one patient for whom CTA images were unavailable and three patients due to image artifacts. Seven patients were also excluded because the proposed semi-automatic reconstruction method was not applicable due to the tortuous geometry of their vessels. Finally, FEAs were performed on data from 89 patients.

### 3.1 Clinical data analysis

We first analysed demographic data in patients with vulnerable plaque compared to patients with stable plaque (Table 1). The macroscopic assessment provided by the surgeon revealed the presence of vulnerable plaques in 43 patients; 10 of them are females, and the median age is 74 years. The clinical characteristics of both groups of patients, as reported in Table 2, showed a similar representation of cardiovascular risk factors between the two groups, with no statistically significant differences, except for a prevalence of higher female patients in the group with stable plaques.

**TABLE 2:** Preoperative characteristics of the analysed cohort of patients.

Demographic data	Vulnerable plaque (N = 43p)	Stable plaque (N = 57p)	p-value
Female sex	10p (23%)	23p (40%)	0.072
Age [years]	74.00 (10.00)	75.00 (11.00)	0.286
BMI	25.88 (6.04)	25.45 (5.11)	0.786
Dyslipidemia	36p (84%)	49p (85%)	0.972
CAD	10p (23%)	12p (21%)	0.792
COPD	1p (2%)	3p (5%)	0.458
Diabetes	14p (33%)	18p (32%)	0.935
Cerebrovascular event	6p (14%)	7p (12%)	0.805

p = patients; N stands for the considered sample size. Categorical data are presented as numbers with the corresponding percentages. Continuous data are presented as median (IQR).

### 3.2 Histological data analysis

The univariate analysis of histological features identified some statistically significant predictors for vulnerable plaques, as reported in Table 3. Vulnerable plaques had a greater necrotic core compared to stable plaques (p-value = 0.018).

**TABLE 3:** Comparison of the data derived from the histological analysis between the group of patients with vulnerable plaque and those with stable plaque.

Histological features	Score	Vulnerable plaque (N = 38p)	Stable plaque (N = 51p)	p-value
Atheroma/necrosis	0	1p (3%)	8p (16%)	**0.018**
	1	19p (50%)	29p (56%)	
	2	18p (47%)	14p (28%)	
Fibrous cap thickness	0	6p (16%)	6p (12%)	0.179
	1	11p (29%)	14p (28%)	
	2	10p (26%)	5p (10%)	
	3	11p (29%)	25p (50%)	
Inflammation	0	20p (53%)	35p (69%)	0.124
	1	18p (47%)	16p (31%)	
Cholesterol	0	8p (21%)	24p (47%)	**0.011**
	1	30p (79%)	27p (53%)	
CD68	0	0p (0%)	16p (31%)	**<0.001**
	1	12p (31%)	15p (30%)	
	2	17p (45%)	16p (31%)	
	3	9p (24%)	4p (8%)	
CD163	0	0p (0%)	15p (29%)	**<0.001**
	1	17p (45%)	24p (47%)	
	2	13p (35%)	7p (14%)	
	3	7p (19%)	5p (10%)	

p = patients. N stands for the considered sample size. Data are presented as numbers with the corresponding percentages. Significant p-values are highlighted in bold.

Atherosclerotic plaques classified as vulnerable were also associated with higher presence of cholesterol in the atheroma (p-value = 0.011). Then, the statistical analysis showed that CD163 and CD68 markers were more expressed in vulnerable than in stable plaques (p-value < 0.001). Specifically, vulnerable plaques tended to have higher scores for these markers compared to stable ones.

### 3.3 US data analysis

All US measurements, derived from both QAS and pSWE (q-Elaxto) modalities, are reported in Table 4. Although no one of the QAS parameters reaches statistical significance in group comparison, the distension values tend to be lower in stable plaques (302.00 (173.00) µm vs. 281.00 (210.50) µm, p-value = 0.382). Both the α the β index, defined as stiffness indexes, tend to be higher in stable plaques. Also DC and CC tend to be slightly higher in patients with vulnerable plaques than in those with stable plaque, even if differences are not statistically significant. Finally, the PWV values are instead very similar in the two groups.

**TABLE 4:** Comparison of the data derived from the preoperative US imaging (QAS wall-tracking modality and pSWE) between the group of patients with a vulnerable plaque and those with a stable plaque.

QAS and pSWE parameters	Vulnerable plaque (N = 43)	Stable plaque (N = 57)	p-value
Distension [µm]	302.00 (173.00)	281.00 (210.50)	0.382
Diameter [mm]	9.17 (1.63)	8.46 (1.89)	0.064
DC [kPa^−1^]	0.01 (0.00)	0.01 (0.01)	0.930
CC [mm^2^/kPa]	0.55 (0.38)	0.48 (0.54)	0.304
α	8.67 (4.12)	8.50 (10.30)	0.694
β	17.61 (8.17)	17.32 (20.68)	0.699
PWV [m/s]	10.54 (3.54)	10.64 (6.07)	0.704
YM_plaque_ [kPa]	12.40 (34.50)	34.70 (82.15)	**0.008**
YM_ICA_ [kPa]	6.5 (29.90)	6.00 (39.90)	0.709
YM_CCA_ [kPa]	11.90 (38.40)	5.80 (42.25)	0.335

N stands for the considered sample size. Significant p-values are highlighted in bold.

Concerning q-Elaxto measurements, the YM values show a significant difference between the vulnerable and stable plaque groups when measurements are acquired in the plaque region (12.40 (34.50) kPa vs. 34.70 (82.15) kPa, p-value = 0.008). Instead, the other two measurements performed in regions without plaques (i.e., YM_ICA_ and YM_CCA_) do not show significant differences between the two groups.

### 3.4 Morphological analysis

Results obtained considering the volumes of the different plaque components are listed in Table 5. The patients with stable plaques show the lower presence of calcium components as compared to the vulnerable plaque group (10.47 (12.61) % vs. 25.94 (46.56) %, p-value = 0.003 for relative volumes; 42.41 (65.66) mm3 vs. 101.62 (200.41) mm3, p-value = 0.013 for absolute volumes) and a higher presence of lipidic components (6.06 (14.96) % vs. 0.96 (6.81) %, p-value = 0.005 for relative volumes; 20.50 (69.78) mm3 vs. 2.09 (21.15) mm3, p-value = 0.005 for absolute volumes). Also the volume of the fibrous content shows significant differences between the two groups. The total volume of the plaque is the only volume variable that does not show a significant p-value.

**TABLE 5:** Comparison of volumes (both percentage and absolute values) between the group of patients with vulnerable plaque and those with stable plaque.

Volume	Vulnerable plaque (N = 40)	Stable plaque (N = 49)	p-value
Calcific content [%]	10.47 (12.61)	25.94 (46.56)	**0.003**
Lipid content [%]	6.06 (14.96)	0.96 (6.81)	**0.005**
Fibrous content [%]	73.78 (26.42)	63.35 (39.472)	**0.015**
Calcific content [mm^3^]	42.41 (65.66)	101.62 (200.41)	**0.013**
Lipid content [mm^3^]	20.50 (69.78)	2.09 (21.15)	**0.005**
Fibrous content [mm^3^]	264.09 (489.66)	197.47 (251.83)	0.147
Total plaque [mm^3^]	397.44 (566.09)	376.10 (370.49)	0.692

N stands for the considered sample size. Data are presented as median (IQR). Significant p-values are highlighted in bold.

### 3.5 Biomechanical analysis

As illustrated in Section 2.4.3, VM_99_, VM_mean_, MPS_99_, and MPS_mean_ stress parameters were computed in each plaque component. Thus, Table 6 reports the statistical analysis results showing possible differences for these parameters (evaluated in each component) between the two groups of patients. Particularly, only the VM99 stress calculated in the lipid component is statistically significant (0.32 (0.47) kPa vs. 0.16 (0.33) kPa, p-value = 0.021) among the simulated stress variables in each component of the plaque.

**TABLE 6:** Comparison of stress parameters in each plaque component and global stress indexes between the group of patients with vulnerable plaque and those with stable plaque.

Plaque component	Stress parameter [kPa]	Vulnerable plaque (N = 40)	Stable plaque (N = 49)	p-value
Calcific component	VM_99_	100.51 (77.79)	97.92 (130.63)	0.314
	MPS_99_	177.47 (179.02)	191.36 (235.26)	0.228
	VM_mean_	25.06 (20.24)	28.03 (36.99)	0.306
	MPS_mean_	18.94 (16.48)	18.76 (27.11)	0.314
Lipid component	VM_99_	0.32 (0.47)	0.16 (0.33)	**0.021**
	MPS_99_	0.94 (1.25)	0.62 (1.57)	0.224
	VM_mean_	1.13 (0.16)	0.06 (0.17)	0.062
	MPS_mean_	-0.01 (0.67)	0.00 (0.124)	0.100
Fibrous component	VM_99_	36.21 (19.52)	36.21 (17.07)	0.882
	MPS_99_	73.57 (45.29)	91.33 (49.52)	0.242
	VM_mean_	12.46 (6.38)	11.96 (8.94)	0.547
	MPS_mean_	12.03 (6.21)	11.65 (7.86)	0.711

Stress index [kPa]	Vulnerable plaque (N = 40)	Stable plaque (N = 49)	p-value
VM_99_	42.55 (25.30)	54.89 (45.89)	0.034
MPS_99_	90.92 (43.90)	113.06 (88.25)	0.011
VM_mean_	13.03 (8.87)	16.48 (14.53)	0.063
MPS_mean_	11.64 (8.31)	13.45 (11.23)	0.094

N stands for the considered sample size. Data are presented as median (IQR). Significant p-values are highlighted in bold.

Each stress parameter computed in the single plaque components was characterized by different values. Indeed, the stress associated to calcific components had significantly higher values, while intermediate and lower values were shown by the fibrous and lipid components, respectively.

Moreover, we calculated some global stress indexes relative to the whole plaque, as reported in Section 2.3.3. Only 99-percentile stress indexes showed a statistically significant difference between groups (42.55 (25.30) kPa vs 54.89 (45.89) kPa, p-value = 0.034 for VM_99_; 90.92 (43.90) kPa vs 113.06 (88.25) kPa, p-value = 0.011 for MPS_99_), while mean index values did not.

## 4 Discussion

Our study recruited 100 patients, with carotid plaque stenosis greater than 70%, who were scheduled to undergo CEA. Ultrasound imaging and CTA scans were performed preoperatively; demographic and clinical data were also collected to evaluate the presence of risk factors for plaque vulnerability.

After surgery, the vascular surgeon visually assessed the removed plaques and classified them as either vulnerable or stable.

While no demographic differences were observed between patient groups, it is important to note that there is a prevalence of stable plaques in the female group. Other studies have also indicated gender differences in atherosclerotic populations. Specifically, there are differences for the presence of calcifications, lipid content, and hemorrhages, with a higher prevalence of these components in men compared to women (van Dam-Nolen et al., 2023). Furthermore, the incidence of ischemic stroke varies between men and women, with substantially higher rates in men (van Dam-Nolen et al., 2022).

In addition, histological analysis was performed on post-surgical plaque samples confirming the surgeon’s classification of vulnerable/stable plaques.

Nevertheless, the principal limitation of histological analysis is its invasive nature, as it requires tissue samples that are obtained post-surgery, thereby limiting its applicability in predicting plaque vulnerability during routine clinical practice but could open or be confirmatory to new CTA or US predictive valuations.

Thus, the aim of this study was also to evaluate non-invasive imaging methods (i.e., CTA, US), combined with structural simulations based on FEA, to consider morphological, compositional and also biomechanical aspects of the plaque rupture, in order to have more information for preoperatively identifying patients with plaques that are likely to be vulnerable.

It is worth pointing out that our study uses patient-specific clinical data from different imaging techniques: CTA images were employed for the 3D vessel model creation used for FEA, while US wall tracking modality and elastography were used to analyse other biomechanical factors that could be useful for the assessment of plaque vulnerability.

Specifically, in the comparison of preoperative QAS evaluation data between the two groups, patients with stable, calcified plaques exhibited distension and stiffness-related index values suggesting a tendency towards greater stiffness as compared with those with vulnerable, soft plaques. However, p-values showed no statistically significant differences between QAS data in the two groups. There were instead significant differences in patients’ classification in terms of YM of the CA plaque region. According to other studies (Garrard et al., 2015; Mazzaccaro et al., 2023), a higher YM would indicate the presence of a non-vulnerable plaque.

For what concerns the plaque morphological and stress analyses, we expanded our previous work (Curcio et al., 2023) by involving a greater number of patients. For each patient, the reconstructed atherosclerotic CA model include the three main components of an atheromatous plaque: the calcific and lipid plaque components were segmented from *in vivo* CTA images, whereas the fibrous component and the surrounding healthy wall were reconstructed by means of an *ad hoc* developed procedure (see Supplementary Material).

From our results, the volumes of all three components of the plaque result to be statistically significantly different in the comparison between patients with stable and vulnerable plaques. In agreement with other studies (Ylä-Herttuala et al., 2013; Shi et al., 2020; Sakamoto et al., 2021), a mostly calcified plaque content is usually associated with plaque stability, while mostly lipidic plaques are usually vulnerable. We also analysed the plaque total volume, but it did not show significant differences between the two groups. According to this finding, compared with plaques that cause severe luminal stenosis, vulnerable plaques may cause relatively minor stenosis, although they account for more cases of rupture and thrombosis (Shah, 2003).

After biomechanical simulations were performed for all patients, we analysed the obtained stress distributions within subsets representing each plaque component, for a quantitative evaluation. VM stress and MPS were considered in these analyses, since they are used in several other works in the literature (Gao and Long, 2008; Gao et al., 2009; Leach et al., 2010; Holzapfel, 2014; Mahmoud et al., 2019; Benitez et al., 2021; Bennati et al., 2021). For each plaque component, the maximum stresses were computed by excluding 1% of all elements containing the highest stresses, as done in (Speelman et al., 2008); in addition, also mean stresses were included in our analysis. Furthermore, we investigated the potential of using alternative stress indexes that combine the computed stress parameters and plaque component volumes to distinguish patients with different types of plaque. In our findings, particularly 99-percentile stress indexes can differentiate groups of vulnerable and stable plaques. More specifically, the obtained results show that the VM_99_ and MPS_99_ stresses are higher in stable plaques, according to the material properties assigned to the different structures in the simulations.

Integrating computed stress indices (e.g., VM_99_, MPS_99_) with imaging-derived parameters (e.g., component volumes and stiffness metrics) could indeed enhance the predictive capability of plaque assessment. The YM values, particularly in the plaque region, were significantly lower in vulnerable plaques compared to stable ones. This finding aligns with the lower stiffness of vulnerable plaques due to their compositional differences, including a reduced calcific and fibrous content and increased lipidic components. Similarly, CTA-derived volumetric analyses showed that stable plaques exhibited a greater calcific content, correlating with the increased stiffness, whereas vulnerable plaques demonstrated a higher proportion of lipidic components. Furthermore, biomechanical simulations provided additional evidence by revealing differences in stress distributions across plaque components. Stress indices, such as VM_99_ and MPS_99_, were higher in stable plaques, that had a higher calcific component and stiffness. Thus, these insights on the relationship between biomechanical and imaging-derived data reveal the potential for new non-invasive indices to aid in plaque classification.

The methods employed in this study also show some limitations. First, US acquisitions could present shadowing and reverberation artefacts. Moreover, blood and subcutaneous fat can attenuate wave propagation, which may render the measurements invalid. Additionally, other problems could include the variability introduced by the subjective selection of the ROI for point shear wave elastography and the assumptions about the material under examination to simplify the analysis and the calculated averaged value (Sigrist et al., 2017).

The proposed computational analysis had some limitations too. These included a non-automatic methodology for reconstructing and simulating patient-specific vessel geometries, simplifying assumptions about material properties and residual stresses, as well as the absence of a fluid domain and time-dependent pressure load. Although it is well established that arterial soft tissues are non-linear anisotropic hyperelastic materials, we chose to set the material properties as linear elastic and isotropic in line with other studies in the literature (Gao et al., 2009; Kelly-Arnold et al., 2013; Mahmoud, Hassan, and Mahmoud, 2019; Benitez et al., 2021; Bennati et al., 2021). Additionally, we did not have access to the specific moment in the cardiac cycle of the lumen configuration in CTA scans. This led us to reconstruct CA geometries undefining particular loading conditions such as blood pressure, residual stresses, and axial pre-stretch. However, there are several works that do not account for residual arterial stresses too (Gao et al., 2009; Creane et al., 2010; Leach et al., 2010; Kelly-Arnold et al., 2013; Mahmoud, Hassan, and Mahmoud, 2019; Benitez et al., 2021; Bennati et al., 2021). Although these simplifications could affect the accuracy of our stress simulation results, we aimed to compare computational results from different types of plaques across different patients under the same conditions. Such limitations will be the object of future developments of this work.

Future advancements of our study could also include the development of composite indices that integrate biomechanical and imaging-derived variables for the non-invasive classification of plaques. For example, combining stress parameters obtained from finite element analysis (FEA) with component volumes derived from computed tomography angiography (CTA) and stiffness metrics from ultrasound could provide a more thorough assessment of plaque vulnerability.

In conclusion, the biomechanical variables obtained from US imaging evaluation and FEA, which show significant differences between the two groups with different types of plaque, have the potential to represent indicators of plaque vulnerability. This information can aid in developing a non-invasive risk assessment method for predicting plaque rupture before any potential cerebrovascular event occurs.

## 5 Conclusion

In this work we evaluate the potential of different measures relative to the morphology and composition of CA plaques that are considered promising for preoperatively predicting their vulnerability. Identifying patients with plaques that are likely to be vulnerable represents a crucial step for the risk stratification of patients requiring surgical intervention, as well as for detecting the early stages of unstable plaques, which continues to be a significant challenge. Among the methods analysed, preoperative elastosonography, 3D modelling of the plaque different components and structural biomechanical analysis are valid means to confirm differences between subgroups of patients with vulnerable and stable plaques.

## Data Availability

The raw data supporting the conclusions of this article will be made available by the authors, without undue reservation.
