# Supplementary material for: Biomechanical assessment of vulnerable plaque: from histological evidence to ultrasound elastography and image-based computational patient-specific modelling
Source: Front Bioeng Biotechnol. 2025 Mar 7;13:1478408. doi: 10.3389/fbioe.2025.1478408 (PMC11925902; doi:10.3389/fbioe.2025.1478408)
Supplement: Supplementary file 1 [file Supplementaryfile1.docx]

***Supplementary Material***

**Biomechanical Assessment of Vulnerable Plaque: from Histological Evidence to Ultrasound Elastography and Image-Based Computational Patient-Specific Modelling**

**Nicoletta Curcio^1^, Michele Conti^2,1^, Rosanna Cardani^3^, Laura Valentina Renna^3^, Giacomo Dell’Antonio^4^, Vlasta Bari^5,6^, Giovanni Nano^6,7^, Giulia Matrone^8†^, Daniela Mazzaccaro^7†*^**

*** Correspondence:** Daniela Mazzaccaro: [daniela.mazzaccaro@grupposandonato.it](mailto:daniela.mazzaccaro@grupposandonato.it)

**1. Vessel and Plaque Geometry Reconstruction**

The reconstruction of the atherosclerotic CA wall and plaques starts from the segmentation of the CTA images. The CA lumen and calcific plaque component were segmented using a semi-automatic 3D level-set active contour method, while the lipid plaque component was segmented manually. The segmentation was performed using the ITK-Snap v3.6 software. The accuracy of segmentation was verified by an expert vascular surgeon (D.M.). The surfaces of all the lipid or calcific plaque regions and lumen were exported as STL files.

We decided to model a third component of the plaque, i.e. the fibrous component that identifies the endothelium layer and intimal smooth muscle cells surrounding the calcific and lipidic components. Since fibrous and muscular tissues of the vessel are usually not clearly distinguishable in CTA images, such structures were not segmented, but they were reconstructed as CAD models. We thus developed an ad-hoc procedure to obtain semi-automatically the models of the carotid walls and fibrous part of the plaque. The reconstruction was performed using the Rhinoceros v6.13 software (McNeel and Associates, Seattle, WA, USA) integrated with the Grasshopper v1.0 plug-in.

Firstly, the STL files of plaques and lumen were cut with evenly spaced cross-sectional planes to get the contour curves of each structure (as shown in Figure 1 (a) on the left). Secondly, the vessel wall inner surface was generated by means of the lofting and Boolean operations on lumen curves. Thirdly, the vessel wall outer surface was generated by processing curves of both lumen and plaque components. Specifically, the outer curves of the vessel were obtained from the lumen curves by enlarging these latter. The enlarging procedure was performed by radially shifting the points that define the luminal profile curves and the plaque sections. When both lumen and plaque contour curves were obtained for each cross-sectional plane, they were “merged” in a single curve and then enlarged as well (as shown in Figure 1 (a) on the right). After the extraction of all curves, we removed those sections where the outer wall curves, obtained after the enlargement procedure, showed a narrowing (see Figure 1 (b)), interpolating only the remaining profile curves that model the ideal physiological outer CA wall. The solid geometry of the atherosclerotic wall was obtained by Boolean difference between inner and outer wall surfaces (in red in Figure 1 (c)). The final step of the proposed method defines the geometric modelling of the fibrotic component of the plaque within the carotid artery wall. We chose to model the fibrous content of the plaque as the region that extends in the stenotic area between the lumen and the outer vessel wall, and that surrounds the other components of the plaque (shown in yellow in Figure 1 (c)). For this purpose, we applied a Boolean difference between the healthy lumen i.e., the lumen without the region classified as stenotic, and the reconstruction of the pathological lumen.

The proposed method could perform poorly on some carotid arteries, mainly where more complex geometries of the plaque components could be present. For this reason, we excluded patients with a very tortuous geometry of their carotid vessels as reported in Section 3 of the manuscript.

More details about the proposed geometrical reconstruction method of atherosclerotic carotid with different plaque components modelling are described in (Curcio et al. 2023).


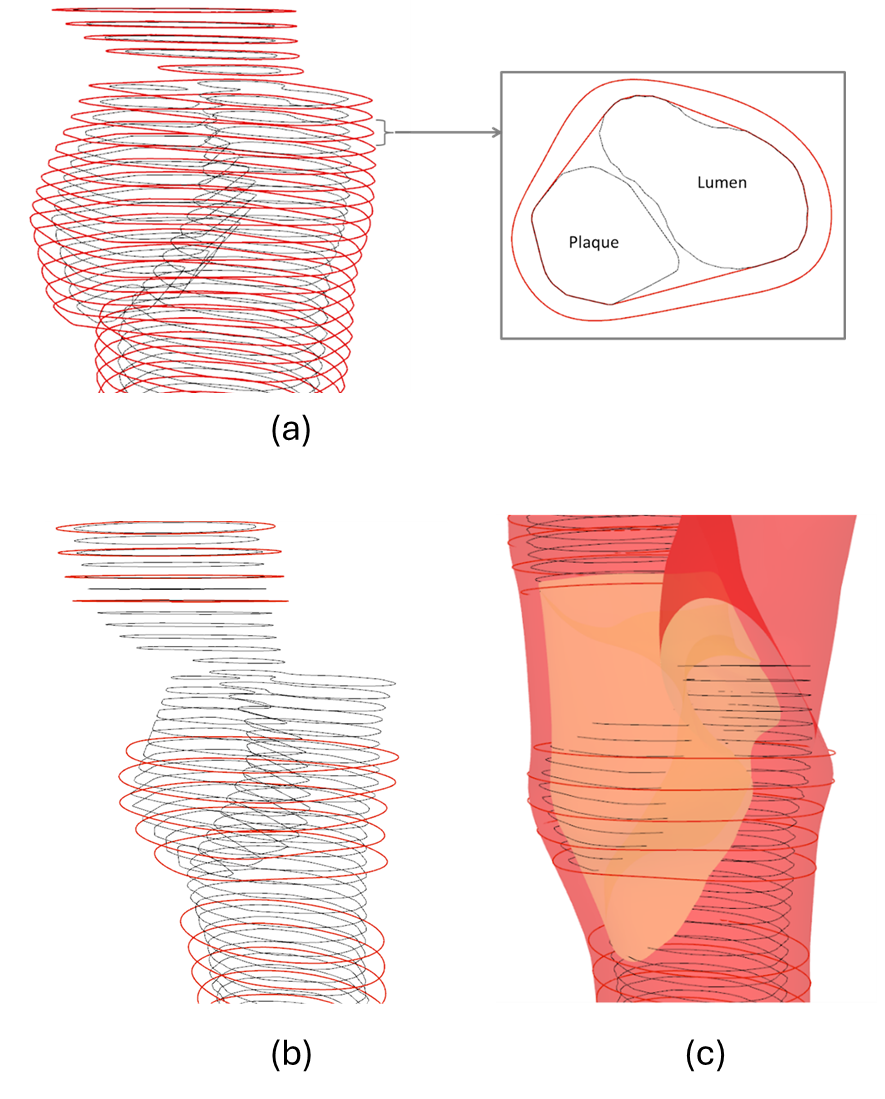


Figure 1. Reconstruction of the atherosclerotic wall and fibrous plaque component models. (a) The generation of the vessel wall outer surface begins with enlarging the lumen contour curves (black curves) and merging them with plaque curves when they appear on the same plane (see the black square). (b) Sections with narrowing outer wall curves are removed, leaving the ideal physiological outer carotid artery wall. (c) The final solid geometry of the atherosclerotic wall (red) is obtained by a Boolean difference between the inner and outer vessel surfaces, while the fibrotic plaque component (yellow) is modeled by a Boolean difference between the ideal healthy and pathological lumen.
